# Toward perturbation theory methods on a quantum computer

**DOI:** 10.1126/sciadv.adg4576

**Published:** 2023-05-12

**Authors:** Junxu Li, Barbara A. Jones, Sabre Kais

**Affiliations:** ^1^ Department of Chemistry, Department of Physics and Astronomy, and Purdue Quantum Science and Engineering Institute, Purdue University, West Lafayette, IN 47907, USA.; ^2^ Department of Physics, College of Sciences, Northeastern University, Shenyang 110819, China.; ^3^ IBM Quantum, San Jose, CA 95120, USA.

## Abstract

Perturbation theory, used in a wide range of fields, is a powerful tool for approximate solutions to complex problems, starting from the exact solution of a related, simpler problem. Advances in quantum computing, especially over the past several years, provide opportunities for alternatives to classical methods. Here, we present a general quantum circuit estimating both the energy and eigenstates corrections that is far superior to the classical version when estimating second-order energy corrections. We demonstrate our approach as applied to the two-site extended Hubbard model. In addition to numerical simulations based on qiskit, results on IBM’s quantum hardware are also presented. Our work offers a general approach to studying complex systems with quantum devices, with no training or optimization process needed to obtain the perturbative terms, which can be generalized to other Hamiltonian systems both in chemistry and physics.

## Teaser

A general quantum circuit for perturbation theory calculations with implementation on IBM’s machines is reported.

## INTRODUCTION

Historically, Schrödinger’s techniques presented in 1926 (*1*) represent the first important application of perturbation theory (PT) for quantum systems, to obtain quantum eigenenergies. With the expansion of theory for atomic and subatomic physics in the first half of the 20th century, PT methods led to a wide variety of applications, such as hyperfine structure (*2*) and the Zeeman (*3*) and Stark effects (*4*). Dirac (*5*), studying the emission and absorption of radiation in 1927, developed a PT result that became Fermi’s golden rule. In quantum field theory, Feynman (*6*) introduced the diagrams known by his name, which represent the perturbative contributions to transition amplitudes. PT is, in addition, a powerful tool for chemists (*7*–*10*). A typical example is Møller-Plesset PT (MPPT) (*11*), where the difference between the exact Hamiltonian and the Hartree-Fock (HF) is included as a perturbation.

We now turn to a brief review of recent advancements in quantum computing. In 2019, Google claimed quantum supremacy with their programmable superconducting processor, progressing on the path to full-scale quantum computing (*12*). In 2020, the quantum computational advantage was once again claimed on a photonic quantum computer (*13*). The fast-paced progress of hardware has resulted in a considerable increase in quantum simulation (*14*–*17*) and error mitigation (*18*) on noisy intermediate-scale quantum (NISQ) devices (*19*). State-of-art variational quantum circuits also attract great interest due to their efficiency and flexibility, leading to a variety of applications ranging from data classification (*20*–*23*) to electronic structure calculations (*24*–*26*). This progress holds the potential for PT methods to be used as an application on quantum devices.

Here, we propose a universal quantum circuit implementation for time-independent PT, or as often termed Rayleigh-Schrödinger PT. Consider the Hamiltonian

$$H=H_{0}+\lambda V$$ (1)

where *H*_0_ is the original Hamiltonian, *V* represents the perturbation, and λ ≪ 1. Denoting the eigenstates and corresponding energy levels of *H*_0_ as $|\psi_{n}^{\left(0\right)}\rangle$ and $E_{n}^{\left(0\right)}$, we have $H_{0}|\psi_{n}^{\left(0\right)}\rangle =E_{n}^{\left(0\right)}|\psi_{n}^{\left(0\right)}\rangle$. Using time-independent PT leads to the following approximation (*27*)

$$E_{n}=E_{n}^{\left(0\right)}+\lambda E_{n}^{\left(1\right)}+\lambda^{2}E_{n}^{\left(2\right)}+O(\lambda^{3})$$

$$|\psi_{n}\rangle =|\psi_{n}^{\left(0\right)}\rangle +\lambda |\psi_{n}^{\left(1\right)}\rangle +O(\lambda^{2})$$

where the first-order correction of eigenstates $|\psi_{n}^{\left(1\right)}\rangle$ and the first- and second-order corrections of energy $\lambda E_{n}^{\left(1,2\right)}$ are included. Mathematically, we have the first-order correction as

$$E_{n}^{\left(1\right)}=\langle \psi_{n}^{\left(0\right)}|V|\psi_{n}^{\left(0\right)}\rangle$$ (2)

$$|\psi_{n}^{\left(1\right)}\rangle =\sum_{m\neq n}\frac{\left\langle \psi_{m}^{\left(0\right)}|V|\psi_{n}^{\left(0\right)}\right\rangle }{E_{n}^{\left(0\right)}-E_{m}^{\left(0\right)}}|\psi_{m}^{\left(0\right)}\rangle$$ (3)

and the second-order correction as

$$E_{n}^{\left(2\right)}=\sum_{m\neq n}\frac{{|\langle \psi_{m}^{\left(0\right)}|V|\psi_{n}^{\left(0\right)}\rangle |}^{2}}{E_{n}^{\left(0\right)}-E_{m}^{\left(0\right)}}$$ (4)

In our approach, simple measurements can be used to estimate the corrections in Eqs. 2 to 4. Because of quantum superposition, the quantum circuit could lead to considerable speedup over classical PT methods. The framework of our method is presented in the “Quantum circuit implementation” and “Application to the extended Hubbard model” sections that demonstrate the design and optimization of the quantum circuit with the extended Hubbard model as an example. In the “Simulation results” section, we present simulation results conducted in Qiskit. The proposed circuit is also implemented on an IBM 27-qubit quantum computer, as presented in the “Implementation on a quantum computer” section. Conclusions and discussions are presented in Discussion. In addition, we present analysis on the scale and time complexity in the “Time complexity” section and further applications in the “Application” section.

## RESULTS

### Quantum circuit implementation

**Fig. 1.**
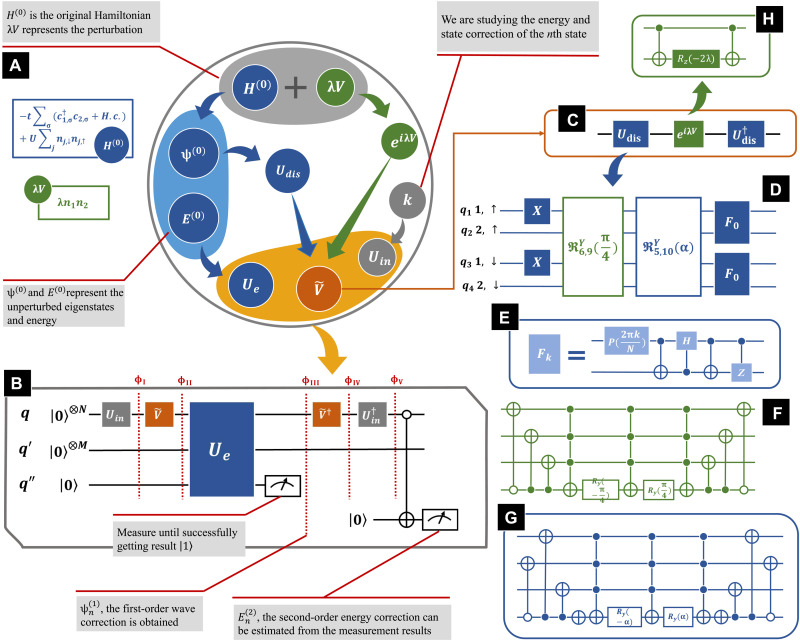
Scheme of the quantum circuit implementation. (**A**) Flowchart of the quantum circuit design process. (**B**) Main structure of the quantum circuit. There are in total *N* + *M* + 2 qubits. The first *N* qubits denoted as *q* represents the system with 2*^N^* basis. The next *M* qubits denoted as *q*′ are included for *U_e_* estimating $1/(E_{n}^{\left(0\right)}-E_{m}^{\left(0\right)})$. The others denoted as *q*′′ are ancilla qubits for readout. $\psi_{n}^{\left(1\right)}$ could be obtained as noted by the dashed line, while $E_{n}^{\left(2\right)}$ would be estimated after measuring the last qubit. (**C**) Structure of $\tilde{V}$, which contains *U*_dis_, $U_{\mathrm{dis}}^{†}$, and exp(*i*λ*V*). (**D**) Structure of *U*_dis_, where *X* represents a NOT gate and *F* indicates Fourier transformation, as shown in (**E**). There are two special multicontrolled rotation gates $R_{6,9}^{y}(\pi /4)$ and $R_{5,10}^{y}(\alpha )$ in *U*_dis_, whose structure can be found in (**F** and **G**). (**H**) Quantum circuit simulating the $\exp(i\lambda \sigma_{z}^{i}\sigma_{z}^{j})$ term.

To estimate the corrections shown in Eqs. 3 and 4 for the *n*th-order terms, there are two important tasks: (i) Estimate the terms of perturbation $\left\langle \psi_{m}^{\left(0\right)}|V|\psi_{n}^{\left(0\right)}\right\rangle$; (ii) estimate the inverse of the energy difference term $1/(E_{n}^{\left(0\right)}-E_{m}^{\left(0\right)})$ for all *m* ≠ *n*. Similarly, there are two main modules in our circuit, an operator denoted $\tilde{V}$ that simulates the perturbation terms and *U_e_* that estimates the inverse of the energy difference. A scheme of the quantum circuit estimating the first-order wave function correction and second-order energy corrections is presented in Fig. 1B. There are in total *N* + *M* + 2 qubits. The first *N* qubits denoted by *q* represent the system with a basis of size 2*^N^*. The next *M* qubits denoted by *q*′ are ancilla qubits, and the last two are included for readout. All qubits are initially prepared in the ground state ∣0〉.

The first step is to initialize the qubits *q* into the general state ∣*k*〉, where ∣*k*〉 indicates the corresponding binary form of the state for which we want to calculate corrections. The gray operator *U_in_* in Fig. 1B represents the initializing process, which generally could be fulfilled with simple NOT gates. For instance, if we would like to study the corrections to the first-excited state, then *U_in_* could be a single NOT gate acting on the last qubit of *q*, converting the qubits in *q* from ground state ∣0〉 (or ∣0…00〉 in binary form) into ∣1〉 (or ∣0…01〉 in binary form). For simplicity, in the following discussion, we denote the quantum states at certain steps as ∣ϕ〉, corresponding to the notations in Fig. 1B. After applying *U_in_*, the qubits are in state ∣ϕ_I_〉 = ∣*k*〉*_q_* ⊗ ∣0〉_*q*′_⊗ ∣0〉_*q*′′_, where the subscripts indicate the corresponding qubits and *k* indicates that we are studying the corrections for the *k*th term.

Next, $\tilde{V}$ is applied on the *q* qubits. The perturbation terms $\left\langle \psi_{m}^{\left(0\right)}|V|\psi_{n}^{\left(0\right)}\right\rangle$ are approximated with $\left\langle m|\tilde{V}|n\right\rangle$. The computational basis terms ∣*n*〉 are often different from the original eigenstates $|{\tilde{\psi }}_{n}^{\left(0\right)}\rangle$ of the unperturbed Hamiltonian *H*^(0)^. Consequently, an additional operator *U*_dis_ is required, which converts the computational basis into the original eigenstates, ensuring that $U_{\mathrm{dis}}|n\rangle =|\psi_{n}^{\left(0\right)}\rangle$. The subscript of *U*_dis_ denotes “disentangle,” and $U_{\mathrm{dis}}^{†}H^{\left(0\right)}U_{\mathrm{dis}}=\sum_{n}E_{n}|n\rangle \langle n|$ is diagonalized under the computational basis. In the “Application to the extended Hubbard model” section, we will present a design of *U*_dis_, especially for the two-site Hubbard model. In addition, a more general quantum circuit implementation of *U*_dis_ for strongly correlated quantum systems can be found in (*28*). If the perturbation *V* can be exactly decomposed into a sequence of unitary operators, then we will have $\tilde{V}=U_{\mathrm{dis}}^{†}VU_{\mathrm{dis}}$. Unfortunately, sometimes, *V* is Hermitian but not unitary. An alternative is to consider exp(*i*λ*V*) as an approximation, as exp(*i*λ*V*) = *I* + *i*λ*V* + O(λ^2^). As shown in Fig. 1C, the more general design is $\tilde{V}=U_{\mathrm{dis}}^{†}\exp(i\lambda V)U_{\mathrm{dis}}$, which guarantees that

$$\left\langle m|\tilde{V}|n\rangle =\delta_{mn}+i\lambda \langle \psi_{m}^{\left(0\right)}|V|\psi_{n}^{\left(0\right)}\rangle +O(\lambda^{2}\right)$$ (5)

Here, the qubits are converted into the state $|\phi_{\mathrm{II}}\rangle ={\left(\sum_{m}\langle m|\tilde{V}|k\rangle |m\rangle \right)}_{q}⊗|0\rangle {}_{q\prime }⊗|0\rangle {}_{q\prime \prime }$, where the state of qubits *q* is rewritten in the computational basis.

*U_e_* (the blue operator in Fig. 1B) is then applied on *q*, *q*′, and *q*′′, generating the inverse of energy difference with

$$\begin{array}{r} U_{e}({|n\rangle }_{q}⊗{|0\rangle }_{q\prime }⊗{|0\rangle }_{q\prime \prime })=\left\{ \begin{array}{cc} {\;|n\rangle }_{q}⊗{|0\rangle }_{q\prime }⊗{|0\rangle }_{q\prime \prime }, & n=k\\ {|n\rangle }_{q}⊗{|0\rangle }_{q\prime }⊗{\left(\sqrt{1-\frac{C^{2}}{{\left(E_{k}-E_{n}\right)}^{2}}}|0\rangle +\frac{C}{E_{k}-E_{n}}|1\rangle \right)}_{q\prime \prime }, & n\neq k \end{array}\right. \end{array}$$ (6)

where *C* is a real constant, ensuring that $0\leq |\frac{C}{E_{k}-E_{n}}|\leq 1$. *U_e_* contains a few multicontroller gates, where the *q* qubits are control qubits and *q*′′ is the target. *U_e_* is determined by the energy levels, and quantum circuit implementation of *U_e_* is a general method. More details of *U_e_* can be found in the “Application to the extended Hubbard model” section. Substituting Eq. 6 into *U_e_* ∣ϕ_II_〉, the output quantum states become

$$\begin{array}{rl} U_{e}|\phi_{\mathrm{II}}\rangle = & \langle k|\tilde{V}|k\rangle {|k\rangle }_{q}⊗{|0\rangle }_{q\prime }⊗{|0\rangle }_{q\prime \prime }\\ & +\sum_{m\neq k}\left\{{|m\rangle }_{q}⊗{|0\rangle }_{q\prime }⊗\left(\langle m|\tilde{V}|k\rangle \sqrt{1-\frac{C^{2}}{{\left(E_{k}-E_{n}\right)}^{2}}}|0\rangle +C\frac{\left\langle m|\tilde{V}|k\right\rangle }{E_{k}-E_{n}}|1\rangle \right)\right\} \end{array}$$ (7)

Here, the repeat-until-success (*29*) strategy is performed as follows. Measure the qubit *q*′′, and if the readout is ∣1〉, then the quantum state will collapse into $|\phi_{\mathrm{III}}\rangle =\sum_{m\neq k}C^{\prime }\frac{\left\langle m|\tilde{V}|k\right\rangle }{E_{k}-E_{n}}{|m\rangle }_{q}⊗{|0\rangle }_{q\prime }⊗{|1\rangle }_{q\prime \prime }$, where *C*${\;}^{\prime }$ is a normalization constant. Otherwise, repeat the whole process above until result ∣1〉 is obtained when measuring *q*′′. Notice that since $\left\langle n|\phi_{\mathrm{III}}\rangle =C^{\prime }\langle \psi_{n}^{\left(0\right)}|\psi_{k}^{\left(1\right)}\right\rangle$, we now successfully get the first-order eigenstate correction. By measuring *q* qubits, we can estimate the first-order eigenstate $|\psi_{k}^{\left(1\right)}\rangle$. If we prefer to do further study of $|\psi_{k}^{\left(1\right)}\rangle$ with a quantum circuit, then ∣ϕ_III_〉 itself is sufficient as an intermediate where the original eigenstates are represented by the corresponding computational basis. For more demanding requirements, $|\psi_{k}^{\left(1\right)}\rangle$ could be obtained after applying *U*_dis_ on the qubits *q*, as $U_{\mathrm{dis}}|\phi_{\mathrm{III}}\rangle =|\psi_{k}^{\left(1\right)}\rangle$.

Since $E_{n}^{\left(2\right)}=\langle \psi_{n}^{\left(0\right)}|V^{†}|\psi_{n}^{\left(1\right)}\rangle$, we can obtain the second-order energy corrections by applying operator ${\tilde{V}}^{†}$ and $U_{in}^{†}$. After applying ${\tilde{V}}^{†}$ on qubits *q* of ∣ϕ_III_〉, we have $|\phi_{\mathrm{IV}}\rangle =\sum_{m\neq k}C^{\prime }\frac{\left\langle m|\tilde{V}|k\right\rangle }{E_{k}-E_{n}}{\tilde{V}}^{†}{|m\rangle }_{q}⊗{|0\rangle }_{q\prime }⊗{|1\rangle }_{q\prime \prime }$. Then, applying $U_{in}^{†}$, we have $|\phi_{V}\rangle =\sum_{m\neq k}C^{\prime }\frac{\left\langle m|\tilde{V}|k\right\rangle }{E_{k}-E_{n}}U_{\mathrm{in}}^{†}{\tilde{V}}^{†}{|m\rangle }_{q}⊗{|0\rangle }_{q\prime }⊗{|1\rangle }_{q\prime \prime }$. Notice that

$$\begin{array}{rl} \left({\langle 0|}_{q}⊗{\langle 0|}_{q\prime }⊗{\langle 1|}_{q\prime \prime })|\phi_{V}\right\rangle & =\sum_{m\neq k}C^{\prime }\frac{\left\langle m|\tilde{V}|k\right\rangle }{E_{k}-E_{n}}\langle 0|U_{in}^{†}{\tilde{V}}^{†}|m\rangle \\ & =\sum_{m\neq k}C^{\prime }\frac{\left\langle m|\tilde{V}|k\right\rangle }{E_{k}-E_{n}}\langle k|{\tilde{V}}^{†}|m\rangle \\ & =\sum_{m\neq k}C^{\prime }\frac{{|\langle m|\tilde{V}|k\rangle |}^{2}}{E_{k}-E_{n}} \end{array}$$ (8)

If we measure all *q* qubits, then the probability to get all at state ∣0〉 will approximate $E_{k}^{\left(2\right)}$. Alternatively, a multicontrolled gate could help reduce the measurement times as shown in Fig. 1B, where an additional ancilla qubit initialized at ∣0〉 is required as the target.

### Application to the extended Hubbard model

In this section, we will demonstrate the details of circuit design with the extended Hubbard model. The Hubbard model is a simple but powerful model of interacting quantum particles in a lattice, which successfully describes the transition between conducting and insulating states (*30*). The Hamiltonian of the two-site Fermi Hubbard Model is given by

$$H_{\mathrm{hub}}=-t\sum_{\sigma }(c_{1,\sigma }^{†}c_{2,\sigma }+c_{2,\sigma }^{†}c_{1,\sigma })+U\sum_{j=1,2}n_{i,↑}n_{i,↓}$$ (9)

where *t* denotes the transfer integral, *U* denotes the on-site interaction, and σ = ↑, ↓ indicates the spin. Depending on the atomic species, more general interactions might occur. A typical example is dipole-dipole interactions induced by polarized dipolar atoms, which is comparatively long-ranged but usually modeled as an interaction between nearest neighbors (*31*, *32*). Adding a dipole-dipole interaction, the Hamiltonian of the extended Hubbard model can be written as (*32*)

$$H=H_{\mathrm{hub}}+W(n_{1,↑}+n_{1,↓})(n_{2,↑}+n_{2,↓})$$ (10)

where *W* parameterizes the amplitude of dipole-dipole interactions. When the dipole-dipole interaction is much weaker compared to the hopping term and the on-site interaction, this model becomes a good candidate for PT methods, where *H*_hub_ is taken as the unperturbed Hamiltonian and the dipole-dipole interaction is regarded as the perturbation. Two qubits are required to simulate a single site (spin up and down), so we need in total of four qubits in *q* to study the two-site extended Hubbard model. The *q* qubits are shown in Fig. 1D, where 1 and 2 indicate the site and ↑ and ↓ indicate the spin. For simplicity, here, we study the corrections to the ground state, so that we have *k* = 0 and *U_in_* = *I* is the identity operator.

In Fig. 1A, we present a flowchart illustrating how to design an appropriate quantum circuit studying the given system with PT methods. We start with the unperturbed Hamiltonian *H*^(0)^ and the perturbation term λ*V*, where λ ≪ 1. The first step is to derive the eigenenergy $E_{n}^{\left(0\right)}$ and corresponding eigenstates $\psi_{n}^{\left(0\right)}$ of *H*^(0)^. *H*_hub_ being a typical model well-developed in the past 50 years, the eigenenergies and eigenstates can be regarded as known terms.

With $\psi_{n}^{\left(n\right)}$, we can design *U*_dis_ that converts the computational basis ∣*n*〉 into the corresponding eigenstate, as $U_{\mathrm{dis}}|n\rangle =|\psi_{n}^{\left(0\right)}\rangle$. In addition, we can design *U_e_* generating the inverse of the energy difference. In Fig. 1A, these terms are all colored blue, as the operators *U_e_* and *U*_dis_ are only determined by the unperturbed Hamiltonian *H*^(0)^. That is, provided that a new perturbation is applied on the same *H*^(0)^, these operators could be kept without any changes. Regarding the perturbation term, we consider exp(*i*λ*V*) since the dipole-dipole interaction cannot be decomposed exactly into a sequence of unitary operators. Implementation of these key operators is as follows.

#### Implementation of U_dis_

Figure 1D is a schematic of the operator *U*_dis_. Noticing that a Fourier transform can diagonalize the hopping term $c_{1,\sigma }^{†}c_{2,\sigma }+H.c.$, we apply a quantum Fourier transform (QFT) on *q*_1_ and *q*_2_ (spin up) and *q*_3_ and *q*_4_ (spin down); the construction of the QFT can be found in Fig. 1E, where *P* represents the phase gate. Because of the existence of on-site interactions, QFT itself is not yet sufficient. Two additional operators denoted as ${frakR}_{6,9}^{y}(\pi /4)$ and $R_{5,10}^{y}(\alpha )$ are required, which act as special multicontrolled rotation gates. The matrix form of $R_{5,10}^{y}(\alpha )$ is

$$R_{5,10}^{y}(\alpha )=\begin{array}{rl} & \;\begin{array}{ccccccccc} & 1 & 2 & \;\cdots & \;5 & \;\cdots & \;10 & \;\cdots & 16 \end{array}\\ & \begin{array}{c} 1\\ 2\\ \vdots \\ 5\\ \vdots \\ 10\\ \vdots \\ 16 \end{array}\left(\begin{array}{c} 1\\ 0\\ \vdots \\ 0\\ \vdots \\ 0\\ \vdots \\ 0 \end{array}\;\;\begin{array}{c} 0\\ 1\\ \vdots \\ 0\\ \vdots \\ 0\\ \vdots \\ 0 \end{array}\;\;\;\begin{array}{c} \cdots \\ \cdots \\ \ddots \\ \;\\ \;\\ \;\\ \;\\ \cdots \end{array}\;\begin{array}{c} 0\\ 0\\ \vdots \\ \cos\alpha \\ \vdots \\ \sin\alpha \\ \vdots \\ 0 \end{array}\begin{array}{c} \cdots \\ \cdots \\ \;\\ \;\\ \ddots \\ \;\\ \;\\ \cdots \end{array}\begin{array}{c} 0\\ 0\\ \vdots \\ -\sin\alpha \\ \vdots \\ \cos\alpha \\ \vdots \\ 0 \end{array}\begin{array}{c} \cdots \\ \cdots \\ \;\\ \;\\ \;\\ \;\\ \ddots \\ \cdots \end{array}\;\;\;\begin{array}{c} 0\\ 0\\ \vdots \\ 0\\ \vdots \\ 0\\ \vdots \\ 1 \end{array}\right) \end{array}$$ (11)

where the numbering of the columns and rows indicates the corresponding eigenstates, and

$$\alpha =-2\arccos\left(\frac{2t+\sqrt{U^{2}/4+4t^{2}}}{\sqrt{U^{2}/4+{\left(2t+\sqrt{U^{2}/4+4t^{2}}\right)}^{2}}}\right)$$ (12)

Implementation of these two special operations can be found in Fig. 1 (F and H). In addition, there are two NOT gates applied on *q*_1_ and *q*_3_, which are included to ensure that the state ∣0〉 (or ∣0000〉 in binary form) corresponds to the ground state $|\psi_{0}^{\left(0\right)}\rangle$.

#### Implementation of exp(iλV)

Using Jordan-Wigner transformation (*33*), σ*_z_* = 1 − 2*n*, the perturbation term in Eq. 10 can be written as

$$\lambda V=\frac{W}{4}(\sigma_{1,↑}^{z}\sigma_{2,↑}^{z}+\sigma_{1,↑}^{z}\sigma_{2,↓}^{z}+\sigma_{1,↓}^{z}\sigma_{2,↑}^{z}+\sigma_{1,↓}^{z}\sigma_{2,↓}^{z})+\frac{W}{4}-\frac{W}{2}(n_{1,↑}+n_{2,↑}+n_{1,↓}+n_{2,↓})$$ (13)

In the Hubbard model, the conservation of the total number of particles implies that the last term is a constant, leaving only the first term as the nontrivial component.

For simplicity, we denote λ = *W*/4 ≪ 1. With first-order Trotter decomposition (*34*), we have

$$\exp(i\lambda V)=\exp(i\lambda \sigma_{1,↑}^{z}⊗\sigma_{2,↑}^{z})\exp(i\lambda \sigma_{1,↑}^{z}⊗\sigma_{2,↓}^{z})\exp(i\lambda \sigma_{1,↓}^{z}⊗\sigma_{2,↑}^{z})\exp(i\lambda \sigma_{1,↓}^{z}⊗\sigma_{2,↓}^{z})$$ (14)

The quantum circuit simulating $\exp(i\lambda \sigma_{j}^{z}⊗\sigma_{k}^{z}),(j\neq k)$ is presented in Fig. 1H, and more details can be found in (*35*).

#### Implementation of U_e_

**Fig. 2.**
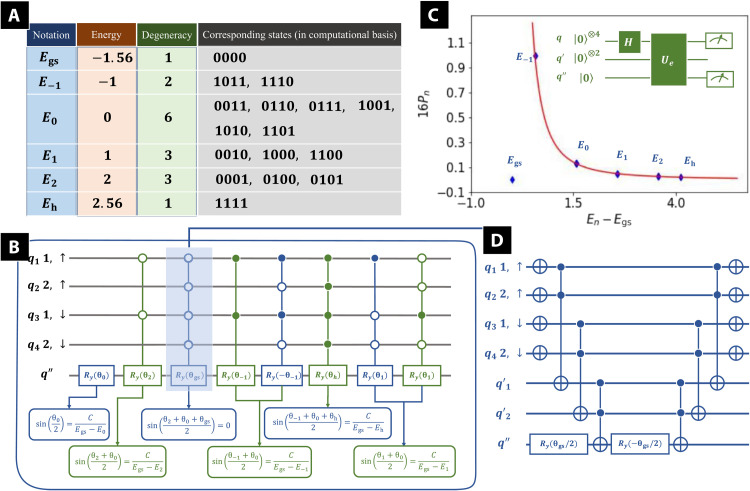
Unperturbed energy levels and implementation of *U_e_*. (**A**) Table of the energy levels, degeneracy, and corresponding states under the computational basis for the unperturbed Hamiltonian *H*^(0)^, where, for simplicity, we set *t* = 1 and *U* = 1. (**B**) Quantum circuit implementation of *U_e_* (the operator *U_e_* in Fig. 1B), where the ancilla qubits *q*′ are not plotted. *U_e_* is constructed with mainly multicontrolled rotation gates. All the parameters θ are determined by the energy levels of the unperturbed Hamiltonian. (**C**) Calibration of *U_e_*. The calibration circuit is plotted in green, shown in the top-right corner. In the calibration circuit, all qubits are initialized as ∣0〉. Hadamard gates are then applied on each *q* qubit, preparing the *q* qubits in a uniform superposition. Then, *U_e_* is applied and qubits *q* and *q*′ are measured. We denote the probability to find the qubits *q* at state ∣*n*〉 and *q*′′ at state ∣1〉 as *P_n_*. The *x* axis denotes the energy difference *E_n_* − *E*_gs_, while the *y* axis denotes 16*P_n_*. (**D**) Decomposition of the multicontrolled rotation gate with light blue background shown in (B) (the second multicontrolled gate from left, colored in blue).

Before discussing the construction of *U_e_*, we need to first calculate the unperturbed energy levels. For simplicity, here, we set *t* = 1 and *U* = 1. Energy levels, degeneracy, and corresponding states under the computational basis for the two-site Hubbard model *H*^(0)^ are presented in Fig. 2A. Here, the eigenstates and eigenenergies are all included (four for the one-electron sector, six for the two-electron sector, four for the three-electron sector, and two trivial terms: four-electron and zero-electron). The ground-state energy is denoted as *E*_gs_, while *E*_h_ represents the energy of the highest excited state. These two states correspond to the ground state and highest excited state of the two-electron Hubbard model (the half-filled case of strong correlations). *E*_0,±1,2_ denotes the other excited-state energies, where the subscripts denote the corresponding energy. Figure 2B is the quantum circuit implementation of *U_e_*, where the ancilla qubits *q*′ are not plotted. *U_e_* is constructed with mainly multicontrolled rotation gates, where the dot on the control qubit indicates that the rotation gate works when this control qubit is ∣1〉 and the circle on the control qubit indicates that the rotation gate works when this control qubit is ∣0〉. First, the energy level *E*_0_ is considered as the “default value,” as it has the most degeneracy. Hence, a simple Ry gate is applied directly on *q*′′, leading to sin(θ_0_/2) = *C*/(*E*_gs_ − *E*_0_), where *C* is the constant in Eq. 6. Then, we study *E*_2_, which contains three degenerate states corresponding to ∣0001〉, ∣0100〉, and ∣0101〉 in the computational basis. Notice that all three states share the same first and third digit as 0, so that a multicontrolled gate with *q*_1_ and *q*_3_ as control qubit is applied, leading to sin[(θ_0_ + θ_2_)/2] = *C*/(*E*_gs_ − *E*_2_). Now, the first multicontrolled rotation gate from left (colored in green) in Fig. 2B is constructed. There is an additional state ∣0000〉 sharing the first and third digits as 0, which corresponds to the ground state. We are now studying the corrections to the ground state, and we need to insure *q*′′ is always at state ∣0〉 when the control qubits are at state ∣0000〉. Therefore, the second multicontrolled rotation gate from left (colored in blue) in Fig. 2B is constructed, ensuring that sin[(θ_0_ + θ_2_ + θ_gs_)/2] = 0. The decomposition of this multicontrolled gate is presented in Fig. 2D, which contains only NOT gates, single-qubit Ry gates, and Toffoli gates. Similarly, the other multicontrolled gates can be constructed, with the corresponding parameters θ determined by the energy levels. In Fig. 2C, we present the calibration of *U_e_*.

The calibration circuit is plotted in green, shown in the top-right corner. In the calibration circuit, all qubits are initialized as ∣0〉. Hadamard gates are then applied on each *q* qubit, preparing the *q* qubits in a uniform superposition. Then, *U_e_* is applied and qubits *q* and *q*′ are measured. Let *P_n_* denote the probability of finding the qubits *q* in state ∣*n*〉 and *q*′′ in state ∣1〉.

Theoretically, $P_{n}=\frac{1}{16}\cdot \frac{C^{2}}{{\left(E_{\mathrm{gs}}-E_{n}\right)}^{2}}$. The *x* axis represents the energy difference *E_n_* − *E*_gs_, while the *y* axis denotes 16*P_n_*. The red curve represents the ideal result, while the blue dots are simulation results for each energy level.

Here, we set *C* = 1/(*E*_gs_ − *E*_−1_), so that *P*_−1_ = 1 reaches the maximum. Calibration results in Fig. 2C prove the ability of *U_e_* shown in Fig. 2B to generate the inverse of energy differences.

### Simulation results

**Fig. 3.**
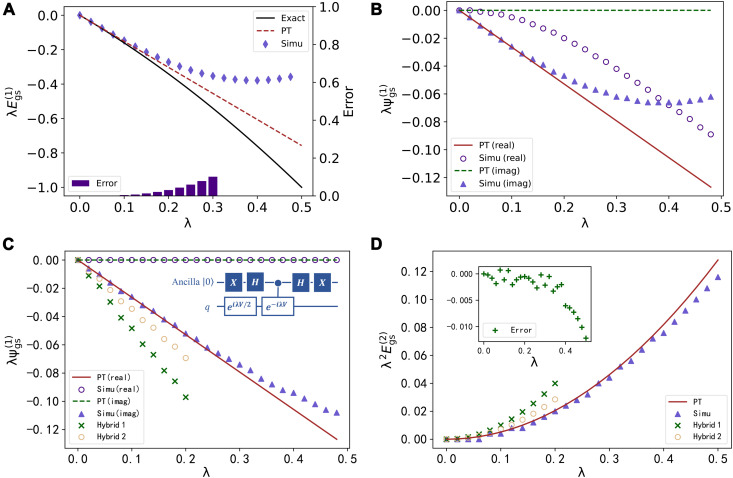
Simulation results, experiment results, and the corresponding prediction by PT methods. All the simulations are performed with Qiskit, while the experiments are implemented on ibmq_montreal. (**A**) First-order energy correction $\lambda E_{\mathrm{gs}}^{\left(1\right)}$. The black curve represents the exact energy change $E_{\mathrm{gs}}-E_{\mathrm{gs}}^{\left(0\right)}$, while the brown dashed line is the first-order energy correction $\lambda E_{\mathrm{gs}}^{\left(1\right)}$. The purple markers denote the estimation of $\lambda E_{\mathrm{gs}}^{\left(1\right)}$ with the quantum circuit. Purple bars at the bottom denote the error between the first-order PT energy correction $\lambda E_{\mathrm{gs}}^{\left(1\right)}$ and the estimation with the quantum circuit. (**B** and **C**) First-order eigenstate correction $\lambda |\psi_{\mathrm{gs}}^{\left(1\right)}\rangle$. The brown lines denote the prediction based on PT methods (solid line for the real part and dashed line for the imaginary part). Purple markers denote the estimation with the quantum circuit (triangles for the imaginary part and circles for the real part). The improved circuit is shown in the top right of (C). (**D**) Second-order energy correction $\lambda^{2}E_{\mathrm{gs}}^{\left(1\right)}$. The brown curve represents the prediction of PT, while the purple triangles denote the estimation with the quantum circuit. The error between the quantum estimation and the PT prediction is presented in the top left of (D). In (C) and (D), the PT corrections estimated from the hybrid calculations are also presented, as marked with green cross symbols (hybrid 1) and orange circles (hybrid 2). In hybrid 1, we applied the full steps of *U_e_*, but in hybrid 2, we only kept the main terms with minimum multicontroller gates.

We studied the first- and second-order energy corrections and first-order eigenstate correction for the ground state of the extended Hubbard model as shown in Eqs. 9 and 10, where we set *t* = 1 and *U* = 1 for simplicity and the simulation results performed on Qiskit are presented in Fig. 3.

Figure 3A shows the approximation of first-order energy correction $\lambda E_{\mathrm{gs}}^{\left(1\right)}$. According to Eq. 5, we have $Im(\langle n|\tilde{V}|n\rangle )=\lambda E_{n}^{\left(1\right)}+O(\lambda^{2})$. Thus, the first-order energy correction can be approximated by the estimation of $Im(\langle m|\tilde{V}|n\rangle )$, requiring only the *q* qubits implementing the operator $\tilde{V}$. In Fig. 3A, the black curve represents the exact energy change $E_{\mathrm{gs}}-E_{\mathrm{gs}}^{\left(0\right)}$, while the brown dashed line is the first-order energy correction $\lambda E_{\mathrm{gs}}^{\left(1\right)}$. The purple markers denote the estimation of $\lambda E_{\mathrm{gs}}^{\left(1\right)}$ with the quantum circuit. The purple bars at the bottom denote the error between the first-order PT energy correction $\lambda E_{\mathrm{gs}}^{\left(1\right)}$ and the estimation with the quantum circuit. For λ < 0.1, the simulation results fit well with the PT prediction, while for greater λ, both the PT prediction and the estimation on the quantum circuit do not do well in approximating the exact energy change.

We show the study of the first-order eigenstate correction $\lambda |\psi_{\mathrm{gs}}^{\left(1\right)}\rangle$ in Fig. 3 (B and C). The brown lines denote the prediction based on PT methods (solid line for the real part and dashed line for the imaginary part). Purple markers denote the estimation with the quantum circuit (triangles for the imaginary part and circles for the real part). In Fig. 3B, we apply exp(*i*λ*V*) to approximate the perturbation. According to Eq. 5, a global phase −*i* is included in the first-order term; thus, the imaginary part of the output will approximate $|\psi_{\mathrm{gs}}^{\left(1\right)}\rangle$. For λ < 0.1, the simulation results fit well with the PT prediction, while for greater λ, the real part of the simulation result increases rapidly, which corresponds to the λ^2^ term in Eq. 5. To approximate the perturbation and eliminate the λ^2^ term, we apply exp(*i*λ*V*/2) − exp(−*i*λ*V*/2) as shown in Fig. 3C. The improved circuit can be found in the top-right corner of the same figure.

Assume that the *q* qubits are initially prepared at ∣ψ_input_〉. If the ancilla qubit is measured and the result is ∣1〉, then we have the *q* qubits at state [exp(*i*λ*V*/2) − exp(−*i*λ*V*/2)]∣ψ_input_〉.

In Fig. 3D, we study the second-order energy correction $\lambda^{2}E_{\mathrm{gs}}^{\left(2\right)}$. Similarly, exp(*i*λ*V*/2) − exp(−*i*λ*V*/2) is applied to approximate the perturbation. The brown curve represents the prediction of PT, while the purple triangles denote the estimation with the quantum circuit. The error between the quantum estimation and the PT prediction is presented in the top left of Fig. 3D. As exp(*i*λ*V*/2) − exp(−*i*λ*V*/2) is applied to approximate the perturbation, the λ/2 terms instead of λ itself dominate the convergence, so that, in Fig. 3 (C and D), the simulation results fit well with the PT prediction for λ < 0.2. Therefore, we can collect the results for a range of λ values and then derive $|\psi_{\mathrm{gs}}^{\left(1\right)}$, 〉 $E_{\mathrm{gs}}^{\left(2\right)}$ with linear regression methods.

### Implementation on a quantum computer

In addition to the simulation performed in Qiskit, we also implement the proposed circuit on IBM’s quantum hardware. As discussed in the “Application to the extended Hubbard model” section, there are three key operations in our proposed circuit, *U*_dis_, exp(*i*λ*V*), and *U_e_*. Both *U*_dis_ and exp(*i*λ*V*) only act on the first four qubits *q*_1,2,3,4_. With the typical Trotter decomposition (*34*), exp(*i*λ*V*) could be implemented with a few simple CNOT gates and Rz gates. In addition, although there are two complicated gates $R_{6,9}^{y}(\pi /4)$ and $R_{5,10}^{y}(\alpha )$ in *U*_dis_, they could be replaced by a two-qubit Bogoliubov transformation along with QFT, as discussed in (*28*). Implementing *U_e_* on quantum devices during the NISQ era is particularly challenging since it involves multiple multicontroller gates and acts on all of the qubits *q*, *q*′, and *q*′′. In comparison to the other two operations, *U_e_* is considerably more complex to implement.

**Fig. 4.**
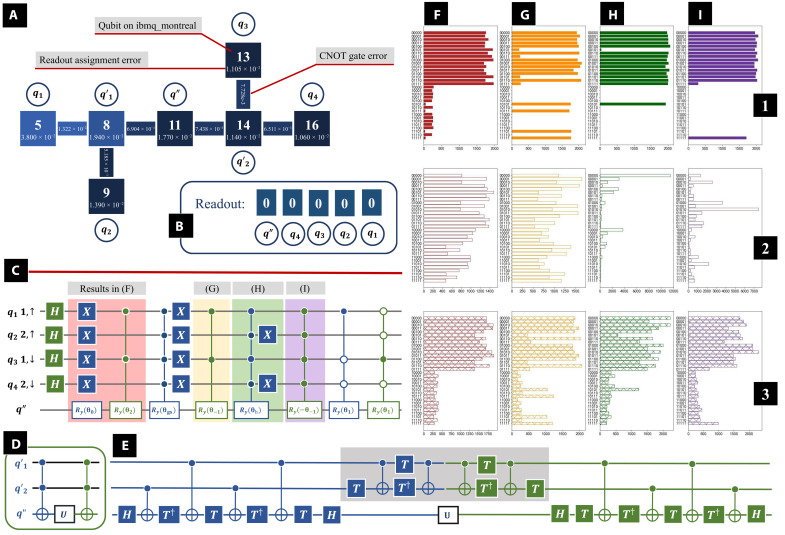
Implementation of *U_e_* on quantum computer ibmq_montreal. (**A**) Qubit mapping on ibmq_montreal, each square indicating a qubit on the quantum computer. (**B**) Relationship between the digits in readout and original qubits. (**C**) Structure of *U_e_*. Here, we study parts of *U_e_* separately, with results of each part presented in (**F** to **I**), where in the first row, we present the simulation results; in the second row, we present the original result; and in the last row, we present the output with improved CCNOT gates pairs, as shown in (D) and (E). (**D**) A pair of CCNOT gates between *q*′_1,2_ and *q*′′ on either side of an operator denoted as *U* acting on the target qubit *q*′′. (**E**) The decomposition of operations is shown in (D). As the operations with gray background cancel out and can be excluded, there is no operation between *q*′_1,2_, which could avoid several SWAP gates in the multicontroller gates.

In this section, we will concentrate on the implementation of *U_e_* on a quantum computer. There are, in total, seven qubits involved in *U_e_*: *q*_1,2,3,4_ representing the physical system, *q*′_1,2_ included to construct the multicontroller gates, and *q*′′ for readout. In Fig. 4C, we present the structure of *U_e_*. Because of *U_e_* being a complicated operation, we study the contribution of the multicontroller gates separately, and the parts of *U_e_* are applied individually. Initially, all qubits are initialized to the ground state ∣0〉. Then, Hadamard gates convert *q*_1,2,3,4_ into a uniform superposition. Next, part of *U_e_* is applied, and *q*_1,2,3,4_ along with *q*′′ are measured at the end, resulting in a binary number. The relationship between the digits in readout and original qubits is presented in Fig. 4B. In particular, here, we present the results of four typical parts in *U_e_*. Two of them mainly contain two-controller rotation gates, which are the operations with background colored in light red and light yellow in Fig. 4C, and the corresponding results are presented in Fig. 4 (F and G). The other two parts mainly contain four-controller rotation gates, as the operations with background colored in light green and light purple in Fig. 4C, whose contribution can be found in Fig. 4 (H and I). In the first row of Fig. 4 (F to I), we present the ideal result without errors, which is obtained from IBM’s simulator, named “simulator_statevector.” There are 32,000 shots in each job (the same as in the following jobs on the real quantum computer). We then ran the parts of *U_e_* on IBM’s 27-qubit quantum computer “ibmq_montreal,” and the results can be found in the second row of Fig. 4 (F to I). At this stage, the bare uncorrected results shown in Fig. 4 (F to I), first row is far different from expectations, with not even the bare shape recognizable.

When a two-qubit operation is performed between the qubits without a physical connection, auxiliary operations such as SWAP gates are required, which unavoidably contribute to additional errors. It is necessary to optimize the qubit mapping by minimizing the required amount of auxiliary operations. Here, we pick seven qubits on ibmq_montreal to implement *U_e_*, as shown in Fig. 4A, where each square indicates a qubit on the quantum computer, the nearby circle infers the qubit mapping, and neighbor qubits are connected. The readout assignment error of each qubit is presented at the bottom of each square, while the CNOT gate error is presented on the bar connecting the squares. The errors usually change after calibration. When we ran the jobs, the median CNOT gate error of ibmq_montreal was 8.636 × 10^−3^, and the median readout error is 1.410 × 10^−2^.

As presented in Fig. 2D, the four-controller rotation gate can be decomposed into CCNOT gates (Toffoli gates) and single-qubit rotation gates. In Fig. 4D, we plot a pair of CCNOT gates between *q*′_1,2_ and *q*′′, bracketing the operator denoted as *U* acting on the target qubit *q*′′. The left CCNOT gate (colored in blue) can be decomposed into several single-qubit gates and CNOT gates, as shown in the left part (colored in blue) of Fig. 4E. In the original decomposition, there are not only CNOT gates between *q*′_1_ and *q*′′ and *q*′_2_ and *q*′′ but also CNOT gates between *q*′_1_ and *q*′_2_. However, on the quantum computer, there is no direct connection between *q*′_1_ and *q*′_2_ as shown in Fig. 4A, and several quantum SWAP gates are required, which can lead to considerable error as shown in the second row of Fig. 4 (F to I). Luckily, the inverse of a CCNOT gate is itself, so we can decompose the other CCNOT gate as the inverse, as shown in the right part (colored in green) of Fig. 4E. Notice that the operations with light gray background cancel out and can be excluded, and there are no more CNOT gates between *q*′_1,2_. Similarly, we can decompose the other CCNOT gate pairs in Fig. 2D.

In the final decomposition of the four-controller rotation gate, there are only CNOT gates between the neighbors, and no auxiliary SWAP gate is required. On the other hand, the two-controller rotation gate can be decomposed into four CNOT gates, two CCNOT gates, and two single-qubit rotation gates (similar to the decomposition in Fig. 2D but replace the first two and last two CCNOT gates with CNOT gates), where no auxiliary SWAP gate is required either.

In the third row of Fig. 4 (F to I) , we present the results on ibmq_montreal with the improved qubit mapping techniques. In addition, because of the degeneracy in unperturbed energy levels, it is possible to reduce the number of multicontroller gates in *U_e_*. In general, *U_e_*, as shown in Fig. 4C, is equivalent to decomposition with one Ry gate, four CRy gates, six CCRy gates, four CCCRy gates, and one CCCCRy gate (a CRy gate contains one controller qubit, a CCRy gate contains two, and so on), some of which could be excluded because of the degeneracy in unperturbed energy levels. Moreover, when studying *U_e_* on the quantum computer, we notice that some multicontroller Ry gates with a small parameter are extremely sensitive and the magnitude of their contribution is less than their average error. In our experiment, multicontroller gates with θ_gs_ and θ_1_ lead to more error than contribute to the overall result. In the Supplementary Materials, a detailed discussion of *U_e_* can be found.

We estimate the PT corrections with a hybrid calculation. The contributions of *U_e_* are estimated on the quantum computer separately, while the operations *U*_dis_ and exp(*i*λ*V*) are estimated via the simulator classically. In Fig. 3 (C and D), we present the PT corrections estimated from the hybrid calculations, as marked with green cross symbols (hybrid 1) and the orange circles (hybrid 2). In hybrid 1, we applied the full steps of *U_e_*, but in hybrid 2, we only kept the main terms with minimum multicontroller gates (terms with small magnitude/large error θ_gs_ and θ_1_ are excluded). Compared with hybrid 1, hybrid 2 results are much closer to the simulation results. The first-order eigenstate correction is estimated from the measurement results of *q* and *q*′′, containing the contributions of both the imaginary part and the real part. Here, we concentrate on the PT eigenstate and eigenenergy corrections for the ground state, where the main contribution is from the term proportional to $\frac{1}{E_{h}-E_{\mathrm{gs}}}$, where *E*_h_ is the state with the highest energy. As shown in Fig. 2C, $\frac{1}{E_{h}-E_{\mathrm{gs}}}$ is the minimum among all such energy difference–dependent terms.

Consequently, the corresponding output in *U_e_* is quite small and sensitive to the existence of errors. Thus, the PT corrections from both hybrid calculations are much greater than the simulation results, which are marked with purple triangles in Fig. 3 (C and D). Even more accurate results could be obtained with state-of-the-art quantum error mitigation techniques (*36*, *37*).

## DISCUSSION

In conclusion, we propose a general quantum circuit estimating both the energy and eigenstates corrections with PT. The quantum approach is demonstrated with application to the two-site extended Hubbard model, where we present numerical simulations based on Qiskit. Furthermore, we implement the proposed circuit on the IBM 27-qubit quantum computer, ibmq_montreal, demonstrating the practicality of estimating PT corrections with quantum hardware. Compared to classical PT, the quantum method is always more efficient in estimating the second-order energy correction $E_{n}^{\left(2\right)}$ for complex systems. When studying complex systems with considerable degeneracy, the quantum method is also more efficient in estimating the first-order eigenstate correction $|\psi_{n}^{\left(1\right)}\rangle$. Moreover, all parameters in the quantum circuit are determined directly by the given Hamiltonian, eliminating any training or optimization process. Our work provides a new approach to studying complex systems with quantum devices, making it possible to implement PT-based methods on with a quantum computer on a wide variety of problems in chemistry and physics.

## MATERIALS AND METHODS

### Time complexity

In this section, we will briefly analyze the time complexity of our method and compare it with classical PT. The unperturbed energy and eigenstates are always required in PT methods. When the unperturbed Hamiltonian *H*_0_ is not available or hard to compute, the popular quantum variational circuit would be a better choice. Here, we assume that the unperturbed Hamiltonian is already well studied so that $E_{n}^{\left(0\right)}$, $|\psi_{n}^{\left(0\right)}\rangle$ are given initially, and the time complexity to derive $E_{n}^{\left(0\right)}$ and $|\psi_{n}^{\left(0\right)}\rangle$ is not included in the following discussion.

Consider a system with 2*^N^* basis states and *L* different energy levels, where *L* ≤ 2*^N^*. Because of the existence of degeneracy, *L* can be sometimes much less than the number of basis states. One example can be found in Fig. 2A, where there are 16 basis states but only 6 different energy levels. Referring to the quantum circuit shown in Fig. 1, we need *N* qubits representing the system with 2*^N^* basis states. Here, we studied the extended Hubbard model, which contains on-site energy and interactions between nearest neighbors, leading to O(*N*) time complexity simulating the perturbation *V* or exp(*i*λ*V*). As for more complicated systems with long-range interactions, theoretically, no more than O(*N*^2^) would be required to simulate the perturbation. Assuming interactions between nearest and next-nearest neighbor sites in an *N*-site model, the number of pairs of sites is *N*(*N* − 1)/2. Simulating each interaction would require multiple two-qubit gates, resulting in an overall time complexity of no more than O(*N*^2^) for simulating these long-range interactions.

In the study of the extended Hubbard model, we construct the operator *U*_dis_ with QFT on the nearest neighbors and two special multicontrolled rotation gates. The Fourier transform part requires O(*N*) time complexity, while the multicontrolled rotation gates with *N* controlled qubits can be decomposed into O(*N*^2^) CNOT gates and single-qubit rotation gates (*38*), leading to O(*N*^2^) time complexity. Including all of the above, the time complexity to estimate the perturbation terms $\left\langle \psi_{m}^{\left(0\right)}|V|\psi_{n}^{\left(0\right)}\right\rangle$ is no more than O(*N*^2^). To estimate a quantum output within error ϵ, $O\left(\frac{1}{\epsilon^{2}}\right)$ measurement time is required (*39*). In total, the time complexity estimating the first-order energy correction $E_{n}^{\left(1\right)}$ is O(*N*^2^/ϵ^2^). Meanwhile, there are *L* multicontrolled rotation gates in *U_e_*, leading to O(*LN*^2^) time complexity. Therefore, the time complexity estimating the first-order eigenstate correction and second-order energy correction is O(*LN*^2^/ϵ^2^).

In contrast, classical PT estimates the corrections as shown in Eqs. 2 to 4. When estimating the first-order energy correction $E_{n}^{\left(1\right)}$, only one term is calculated. However, O(2*^N^*) terms are calculated to estimate the first-order eigenstate correction $|\psi_{n}^{\left(1\right)}\rangle$, and a further O(4*^N^*) terms are calculated to estimate the second-order energy correction $E_{n}^{\left(2\right)}$. The number of basis states dominates the time complexity of classical PT methods. Compared with classical PT, our quantum version does not show speed up when estimating the first-order energy correction $E_{n}^{\left(1\right)}$. However, our quantum circuit can also generate the quantum state of the first-order eigenstate correction $|\psi_{n}^{\left(1\right)}\rangle$. When studying a complex system with considerable degeneracy, we have *L* ≪ 2*^N^*, and the quantum methods can lead to speedup when estimating $|\psi_{n}^{\left(1\right)}\rangle$. The quantum version leads to speedup when estimating the second-order energy correction $E_{n}^{\left(2\right)}$ of complex systems with large size, since for large *N* values, we have O(*LN*^2^/ϵ^2^) < O(4*^N^*).

### Applications

In the “Application to the extended Hubbard model” and “Implementation on a quantum computer” sections, the proposed quantum circuit design and implementation on real quantum hardware are demonstrated in detail, with application to the extended two-site Hubbard model. In this section, we would like to expand on the class of problems to which our method could be applied.

In addition to the simple two-site Hubbard model, our proposed method is applicable to other strongly correlated quantum systems. There are three key operations in our proposed quantum circuit, *U*_dis_, exp(*i*λ*V*), and *U_e_*. In the “Application to the extended Hubbard model” section, we present a universal design of *U_e_* with multicontroller gates. Similarly, given known perturbation *V*, we could design exp(*i*λ*V*) with Trotter decomposition. Meanwhile, Verstraete *et al*. (*28*) developed the explicit quantum circuits that diagonalize the dynamics of strongly correlated quantum systems with a Bogoliubov transformation and QFT, with which *U*_dis_ could be generalized to these quantum systems. Our proposed quantum circuit therefore could be applied to other strongly correlated quantum systems. As an example, in the Supplementary Materials, we present another application, to a Heisenberg *XY* chain.

Furthermore, as PT is always a powerful tool for chemists solving many quantum chemistry problems, our proposed quantum circuit could also be applied to electronic structure calculations for atoms and molecules. For instance, MPPT (*11*) is a typical post–HF ab initio method in the field of computational chemistry, where an HF calculation is used as the starting point, and the difference between the exact Hamiltonian and the HF one is included as a perturbation. In recent years, we have witnessed a multiplicity of quantum theoretical and experimental tools for the prediction of molecular properties and chemical reactions pathways and structure, especially with the HF method. In 2020, Google AI Quantum successfully obtained the HF wave function for a linear chain of 12 hydrogen atoms with a variational quantum eigensolver (VQE) simulation on their Sycamore quantum processor (*40*). These advances bring us more promising applications, making it possible to develop quantum circuits for MPPT calculations, where the HF results could be obtained from quantum devices with VQE simulation and the PT calculations from our proposed quantum circuit. In summary, the proposed general quantum circuit could be applied to various strongly correlated many-body quantum systems.
