# Supplementary material for: Toward perturbation theory methods on a quantum computer
Source: Sci Adv. 2023 May 12;9(19):eadg4576. doi: 10.1126/sciadv.adg4576 (PMC10181180; doi:10.1126/sciadv.adg4576)
Supplement: Supplementary file 1 — Supplementary Materials Figs. S1 to S4 Table S1 [file sciadv.adg4576_sm.pdf]

Science Advances
AAAS

Supplementary Materials for

Toward perturbation theory methods on a quantum computer

Junxu Li *et al.*

Corresponding author: Sabre Kais, kais@purdue.edu

Sci. Adv. **9**, eadg4576 (2023)
DOI: 10.1126/sciadv.adg4576

This PDF file includes:

Supplementary Materials
Figs. S1 to S4
Table S1

Supplementary Materials

A. An alternative implementation of the main circuit

In the main article, the Repeat-Until-Success(RUS) strategy is performed to obtain the first order eigenstate correction $|\psi_n^{(1)}\rangle$, which is then used for the next circuit estimating $E_n^{(2)}$. The RUS process includes the measurement on intermediate states, which raises additional requirements for the experimental apparatus. Sometimes it might be difficult to apply successive operators after intermediate measurements. In this case, we can end at state $|\phi_{III}\rangle$ shown in Fig.(1b), with which the first order eigenstate correction $|\psi_n^{(1)}\rangle$ could be estimated. In Fig.(S1), we present an optional circuit implementation to estimate the second order energy correction $E_n^{(2)}$ without the RUS process. Differing from the original circuit shown in Fig.(1b), q'' is not measured after U_e. Instead, q'' performs as control qubit in the multi-controlled NOT gate at the end. In this way, we could estimate $E_n^{(2)}$ without measurements on the intermediate states.

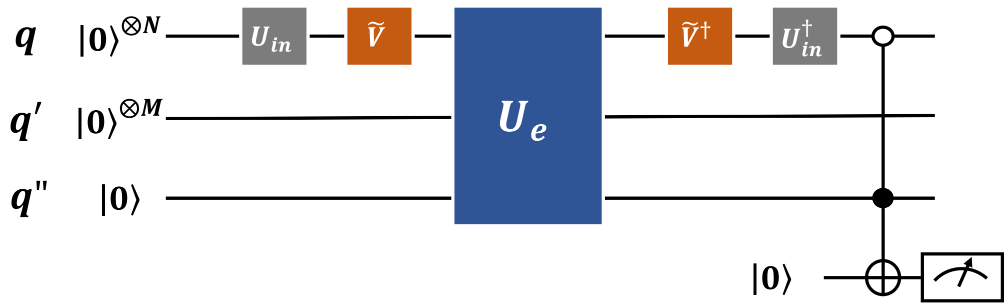

Figure S1: **Scheme of the alternative implementation of the main circuit.**

B. Application to a Heisenberg XY chain

Here we give another application of our quantum circuit for perturbation theory (PT) methods. Consider the Heisenberg XY chain, whose Hamiltonian is described as

$$H = \sum_j \sigma_j^x \sigma_{j+1}^x + \gamma \sum_j \sigma_j^z + \lambda \sum_j \sigma_j^y \sigma_{j+1}^y \tag{S1}$$

where $\gamma \sum_j \sigma_j^z$ represents an external transverse magnetic field. We assume that the YY interaction is much weaker as compared to the XX interaction, so that $\lambda \ll 1$, and the YY interaction terms can be regarded as perturbations.

The decomposition of $\tilde{V}$ can be found in Fig.(S2). As we are studying a new system, the operator U_{dis} and $\exp(i\lambda V)$ are different than the previous Hamiltonian. The structure of U_{dis} is presented in Fig.(S2b), which converts the computational basis $|n\rangle$ into the unperturbed eigenstates $|\psi_n^{(0)}\rangle$, leading to $U_{dis}|n\rangle = |\psi_n^{(0)}\rangle$. Operator B represents a Bogoliubov transformation, while F represents the quantum Fourier transform. The grey box is a fermionic swap gate. The structure of the quantum Fourier transform is shown in Fig.(S2c), while the structure of the Bogoliubov transformation can be found in Fig.(S2d). With Trotter decomposition, operator $\exp(i\lambda V)$ can be decomposed into the ZZ interaction shown in Fig.(S2e) and the interaction at the boundary as shown in Fig.(S2f). More details about the operator U_{dis} for Ising type Hamiltonians can be found in [28, 41].

Once the unperturbed energy levels $E_n^{(0)}$ are given, U_e can be constructed following the method discussed in Sec.(2) of the main article. For simplicity, we will not repeat the whole process.

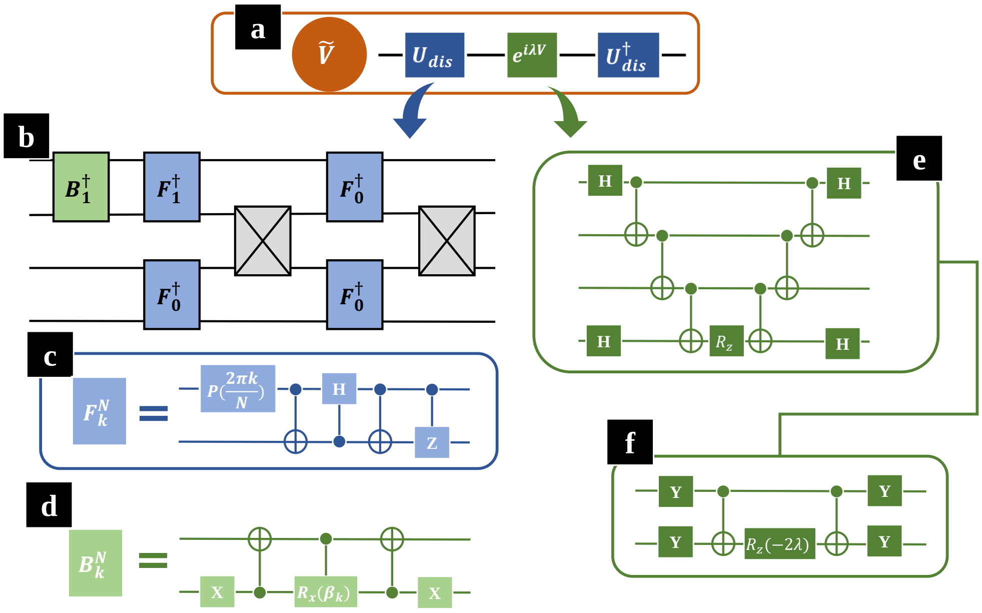

Figure S2: **Scheme of the quantum circuit implementation for the Heisenberg XY chain.** (a.) The decomposition of $\widetilde{V}$. As we are studying a new system, the operator U_{dis} and $\exp(i\lambda V)$ are different than eg Fig.(1D,H). (b.) Structure of U_{dis} that converts the computational basis $|n\rangle$ into the unperturbed eigenstates $|\psi_n^{(0)}\rangle$. Operator B represents a Bogoliubov transformation, while F represents the quantum Fourier transform. The grey box is a fermionic swap gate. (c.) Structure of the quantum Fourier transform. (d.) Structure of the Bogoliubov transformation. With Trotter decomposition, operator $\exp(i\lambda V)$ could be decomposed into the ZZ interaction shown in (e) and the interaction at boundary as shown in (f).

C. More discussion about U_e

In this section, we would like to present more details about U_e. As shown in Fig.(4c), U_e could be decomposed into a few multicontroller gates along with several simple single qubit gates. When implementing the multicontroller gates on a quantum device, we apply the decomposition as shown in Fig.(S3b), which corresponds to the multicontroller gate with green background as shown in Fig.(4c). $q'_{1,2}$ are introduced to connect control qubits $q_{1,2,3,4}$ with the target q". There are CCNOT gates (or Toffoli gates) applied among q_1, q_2 and q'_1, similarly q_3, q_4 and q'_2. Then a CCRy gate is applied among $q'_{1,2}$ and q", which is decomposed into two CCNOT gates and two Ry gates. Finally, there are two CCNOT gates applied among qubits $q_{1,2,3,4}$ and $q'_{1,2}$, ensuring the later ones are reversed to their initial states. In addition to the decomposition of the multicontroller gate, there are some simple Hadamard gates or NOT gates in Fig.(S3b), which correspond to the same ones in Fig.(4c). The CCNOT gates pairs as shown in Fig.(4c) could be decomposed into the circuit shown in Fig.(4d), where the operations with grey background cancel out. Therefore, in the implementation on real quantum devices as shown in Fig.(S3a), there are no more quantum SWAP gates required. In the experiment on real quantum devices, we notice that the CCNOT gates mainly cause error on the target qubit. For instance, when we test the circuit as shown in Fig.(S3b), all qubits are initialized at ground state $|0\rangle$. Theoretically qubits $q'_{1,2}$ should always be $|0\rangle$ at the end, yet in experiment they are often not. Additional Ry gates can then be added on $q'_{1,2}$, and similarly on q", as in the dashed boxes shown in Fig.(S3b).

In Fig.(2b) and Fig.(4c), the U_e are decomposed into multicontroller gates along with several simple single qubit gates. Due to the degeneracy in our example, only 7 multicontroller gates are required. Here we present an equivalent decomposition of U_e as shown in Fig.(S3), where there are 1 Ry gate, 4 CRy gates, 6 CCRy gates, 4 CCCRy gates and 1 CCCCRy gate (a CRy gate contains one controller qubit, a CCRy gate contains two and so on). The design in Fig.(S3) is also universal, but leads to less time

complexity when there is no degeneracy. In Tab.(1), we present the probabilities to get various results. Consider the quantum circuit shown in Fig.(S3c), and measure $q_{1,2,3,4}$ and q". The first four columns indicate the measurement results of $q_{1,2,3,4}$, and the last column represents the probability to obtain the corresponding results on $q_{1,2,3,4}$ while obtaining result 1 on q".

q_1	q_2	q_3	q_4	Probability to get this result
0	0	0	0	$\frac{1}{16}\sin^2\left(\frac{\alpha_0}{2}\right)$
0	0	0	1	$\frac{1}{16}\sin^2\left(\frac{\alpha_0+\alpha_4}{2}\right)$
0	0	1	0	$\frac{1}{16}\sin^2\left(\frac{\alpha_0+\alpha_3}{2}\right)$
0	0	1	1	$\frac{1}{16}\sin^2\left(\frac{\alpha_0+\alpha_3+\alpha_4+\alpha_{3,4}}{2}\right)$
0	1	0	0	$\frac{1}{16}\sin^2\left(\frac{\alpha_0+\alpha_2}{2}\right)$
0	1	0	1	$\frac{1}{16}\sin^2\left(\frac{\alpha_0+\alpha_2+\alpha_4+\alpha_{2,4}}{2}\right)$
0	1	1	0	$\frac{1}{16}\sin^2\left(\frac{\alpha_0+\alpha_2+\alpha_3+\alpha_{2,3}}{2}\right)$
0	1	1	1	$\frac{1}{16}\sin^2\left(\frac{\alpha_0+\alpha_2+\alpha_3+\alpha_4+\alpha_{2,3}+\alpha_{2,4}+\alpha_{3,4}+\alpha_{2,3,4}}{2}\right)$
1	0	0	0	$\frac{1}{16}\sin^2\left(\frac{\alpha_0+\alpha_1}{2}\right)$
1	0	0	1	$\frac{1}{16}\sin^2\left(\frac{\alpha_0+\alpha_1+\alpha_4+\alpha_{1,4}}{2}\right)$
1	0	1	0	$\frac{1}{16}\sin^2\left(\frac{\alpha_0+\alpha_1+\alpha_3+\alpha_{1,3}}{2}\right)$
1	0	1	1	$\frac{1}{16}\sin^2\left(\frac{\alpha_0+\alpha_1+\alpha_3+\alpha_4+\alpha_{1,3}+\alpha_{1,4}+\alpha_{3,4}+\alpha_{1,3,4}}{2}\right)$
1	1	0	0	$\frac{1}{16}\sin^2\left(\frac{\alpha_0+\alpha_1+\alpha_2+\alpha_{1,2}}{2}\right)$
1	1	0	1	$\frac{1}{16}\sin^2\left(\frac{\alpha_0+\alpha_1+\alpha_2+\alpha_4+\alpha_{1,2}+\alpha_{1,4}+\alpha_{2,4}+\alpha_{1,2,4}}{2}\right)$
1	1	1	0	$\frac{1}{16}\sin^2\left(\frac{\alpha_0+\alpha_1+\alpha_2+\alpha_3+\alpha_{1,2}+\alpha_{1,3}+\alpha_{2,3}+\alpha_{1,2,3}}{2}\right)$
1	1	1	1	$\frac{1}{16}\sin^2\left(\frac{\alpha_0+\alpha_{1,2,3,4}}{2}+\frac{1}{2}\sum_a\alpha_a+\frac{1}{2}\sum_{a,b}\alpha_{a,b}+\frac{1}{2}\sum_{a,b,c}\alpha_{a,b,c}\right)$

Table 1: **Probabilities to get various results.** Consider the quantum circuit shown in Fig.(S3c), and measure $q_{1,2,3,4}$ and q". The first four columns indicate the measurement results of $q_{1,2,3,4}$, and the last column represents the probability to obtain the corresponding results on $q_{1,2,3,4}$ while obtaining result 1 on q".

Additionally, due to the existence of multiple sources of noise, we can further reduce the number of multicontroller gates. Some multicontroller Ry gates with small parameters are extremely sensitive, and their contribution is even less than their average error. In our experiment, multicontroller gates with θ_{gs} and θ_1 lead to more error than contribution in the overall result, and we can get a better result after excluding these gates, as shown in Fig.(3c,d).

Another construction of U_e might be useful in certain cases, where the terms are approximated with Fourier series,

$$\frac{C}{E_0-E_n}=\sum_{m=1} a_m\cos(mn+b_m) \tag{S2}$$

The parameters $\{a_m, b_m\}$ guarantee that Eq.(S2) works for all possible n (when $n=0$ the left part is set to 0). In our recent work[42] we present a quantum circuit estimating the Fourier series as shown in Eq.(S2), which would be helpful especially when $\frac{C}{E_0-E_n}$ is periodic with degeneracies.

D. Detailed quantum circuit

In Fig.(S4) we present the detailed quantum circuit estimating the first order eigenstate correction $\psi_0^{(1)}$ for the ground state. The first 4 qubits q_1, q_2, q_3, q_4 represent the system we are studying. q_1', q_2' are

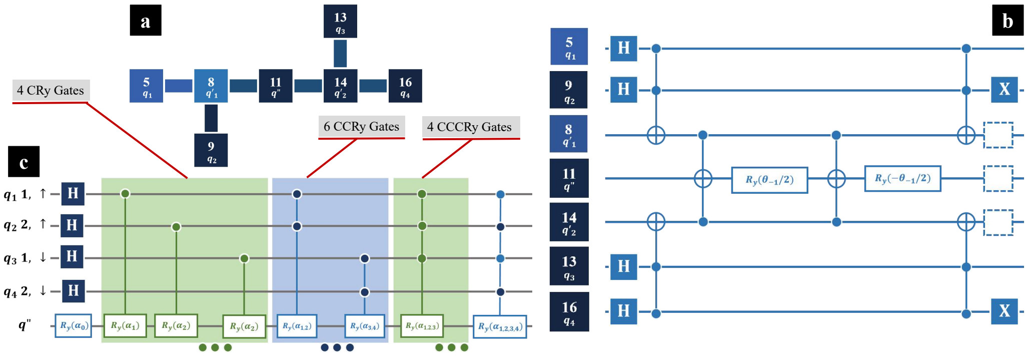

Figure S3: **Details of U_e.** (a) The mapping on quantum computer 'ibmq_montreal', each square indicating a qubit on the quantum computer. (b) Decomposition of the multicontroller gate with 4 control qubits, along with the Hadamard gates on $q_{1,2,3,4}$, and NOT gates on $q_{2,4}$, corresponding to the gate with green background in Fig.(4c) (Step 3). (c) An equivalent decomposition of U_e, where there are 1 Ry gate, 4 CRy gates, 6 CCRy gates, 4 CCCRy gates and 1 CCCCRy gate (a CRy gate contains one controller qubit, a CCRy gate contains two and so on). The Hadamard gates on $q_{1,2,3,4}$ are included to test the performance.

included to construct multicontrolled rotation gates in U_e. $q"_1$ corresponds to the $q"$ shown in Fig.(1b). $q"_2$ is the ancilla qubit included in the improved circuit estimating perturbation, as shown in Fig.(3c). After the whole operation, $q"_1$ is measured. If result $|1\rangle$ is obtained, the first order eigenstate correction could be estimated as discussed in Sec.(1) in the main article. In Fig.(S4), we set $\lambda = 0.1$. In total, there are more than 80 single qubit gates, around 50 two qubit gates (CNOT, CRy, and SWAP gates), 38 Toffoli gates (also CCNOT gates) and 12 CCCNOT gates. All the key operations U_{dis}, U_e, and $\exp(i\lambda V/2) - \exp(-i\lambda V/2)$ are included in the quantum circuit estimating the first order eigenstate correction.

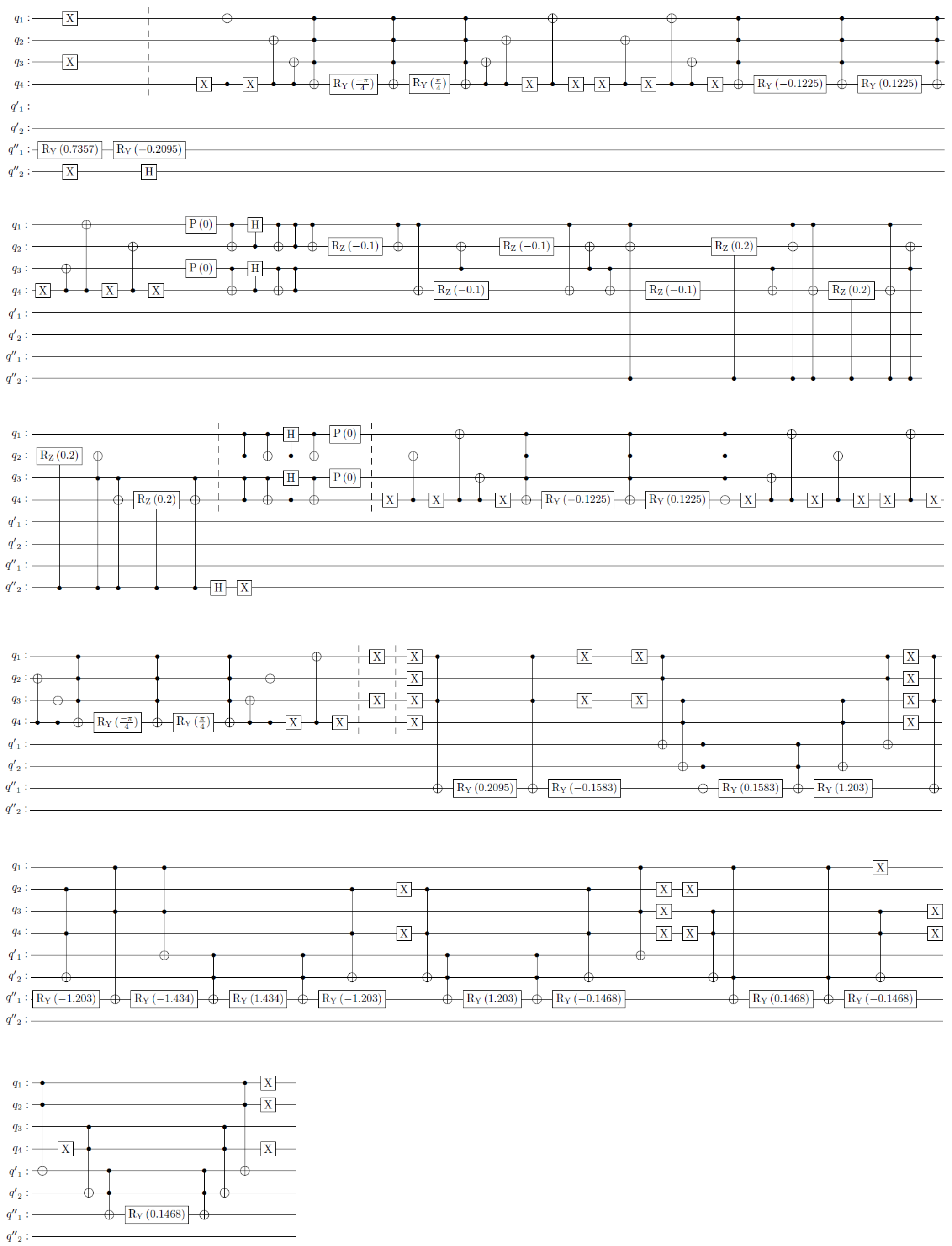

Figure S4: **Scheme of the full quantum circuit estimating the first order eigenstate correction.** The first 4 qubits q_1, q_2, q_3, q_4 represent the system we are studying. q_1', q_2' are included to construct multi controlled rotation gates in U_e. $q"_1$ corresponds to the $q"$ shown in Fig.(1b). $q"_2$ is the ancilla qubit included in the improved circuit estimating perturbation, as shown in Fig.(3c). Here we set $\lambda = 0.1$. In total, there are more than 80 single qubit gates, around 50 two qubit gates (CNOT, CRy, and SWAP gates), 38 Toffoli gates (also CCNOT gates) and 12 CCCNOT gates.
